# Receiver phase alignment using fitted SVD derived sensitivities from routine prescans

**DOI:** 10.1371/journal.pone.0256700

**Published:** 2021-08-30

**Authors:** Olivia W. Stanley, Ravi S. Menon, L. Martyn Klassen

**Affiliations:** 1 Centre for Functional and Metabolic Mapping, The University of Western Ontario, London, Ontario, Canada; 2 Department of Medical Biophysics, The University of Western Ontario, London, Ontario, Canada; University of Minnesota, UNITED STATES

## Abstract

Magnetic resonance imaging radio frequency arrays are composed of multiple receive coils that have their signals combined to form an image. Combination requires an estimate of the radio frequency coil sensitivities to align signal phases and prevent destructive interference. At lower fields this can be accomplished using a uniform physical reference coil. However, at higher fields, uniform volume coils are lacking and, when available, suffer from regions of low receive sensitivity that result in poor sensitivity estimation and combination. Several approaches exist that do not require a physical reference coil but require manual intervention, specific prescans, or must be completed post-acquisition. This makes these methods impractical for large multi-volume datasets such as those collected for novel types of functional MRI or quantitative susceptibility mapping, where magnitude and phase are important. This pilot study proposes a fitted SVD method which utilizes existing combination methods to create a phase sensitive combination method targeted at large multi-volume datasets. This method uses any multi-image prescan to calculate the relative receive sensitivities using voxel-wise singular value decomposition. These relative sensitivities are fitted to the solid harmonics using an iterative least squares fitting algorithm. Fits of the relative sensitivities are used to align the phases of the receive coils and improve combination in subsequent acquisitions during the imaging session. This method is compared against existing approaches in the human brain at 7 Tesla by examining the combined data for the presence of singularities and changes in phase signal-to-noise ratio. Two additional applications of the method are also explored, using the fitted SVD method in an asymmetrical coil and in a case with subject motion. The fitted SVD method produces singularity-free images and recovers between 95–100% of the phase signal-to-noise ratio depending on the prescan data resolution. Using solid harmonic fitting to interpolate singular value decomposition derived receive sensitivities from existing prescans allows the fitted SVD method to be used on all acquisitions within a session without increasing exam duration. Our fitted SVD method is able to combine imaging datasets accurately without supervision during online reconstruction.

## Introduction

Using phase as a contrast has been a subject of interest since the development of magnetic resonance imaging (MRI). Conventional applications of MRI phase have included thermometry [1], susceptibility weighted imaging [2], quantitative susceptibility mapping (QSM) [3, 4], and velocity encoding to measure vessel flow [5]. Improvements in MRI technology and techniques has led to increased popularity of these applications, and has also resulted in the development of many novel techniques that use complex data, such as functional MRI (fMRI) analysis [6–8] and the development of functional QSM [9]. These novel functional applications require the collection of large time series datasets where both the magnitude and phase data are analyzed. The current defaults provided by MRI systems are not always optimized for phase datasets and additional coil combination methods may be required [10]. One such example is the default combination for phase fMRI images which is complex sum on many systems, such as the CMRR Multiband EPI sequence on Siemens systems prior to 2017 [11]. The CMRR Multiband EPI sequence on Siemens systems after 2017 is not known to have any of the issues considered in this paper. It is advantageous to generate the phase and magnitude image volumes during reconstruction on the MRI system because exporting the complex data from each individual coil for offline reconstruction can be resource and time consuming. This is particularly true for functional MRI data sets which are routinely large due to their multi-volume nature.

Phase reconstruction is complicated by the use of multi-element receive arrays that are composed of 32, 64 or more radio frequency (RF) coils. Each RF element in these arrays has a complex, spatially varying receive coil sensitivity profile which weights the measured signal of that element. To form an image with optimal signal-to-noise ratio (SNR), RF arrays require accurate receive coil sensitivities during image combination. At lower magnetic fields, relative receive coil sensitivities are typically obtained by using a reference coil or body coil with a spatially homogeneous sensitivity profile [12]. At ultra-high fields, body coils are rarely available, and if they are, they suffer from poor homogeneity [10]. This translates to poor relative complex-valued sensitivity estimates and thus poor combination of phase data. These in turn result in a reduction in SNR and, in the worst case, phase singularities in the combined phase images. Phase singularities can be caused by destructive interference between coils as the magnitude sums to zero and the phase is undefined. These phase singularities cause issues for downstream phase processing such as spatial unwrapping and high pass filtering [10] and can also be mistaken for pathology [4]. A successful coil combination method should not introduce phase singularities into the combined data.

Coil combination in absence of a physical reference coil has many possible solutions that can be organized into two main categories: inline, where combination is done on the MRI system as images are acquired and reconstructed, or offline, where combination occurs post-acquisition after all volumes have been collected and the data is exported off the MRI system. For large multi-volume imaging sets like those used in fMRI or fQSM, fast, robust, and automatic inline combination is essential to an efficient workflow, as data transfer and handling for offline processing becomes prohibitive. Inline combination methods include complex sum, adaptive combine [13], the virtual reference coil (VRC) [14] and the virtual body coil (VBC) [15]. These methods can often experience issues with robustness. Complex sum and adaptive combine create combinations with phase singularities, indicating their poor combination quality. The VRC method is susceptible to error because it relies on calculating phase of the virtual coil relative to a single voxel [14]. If this voxel is poorly selected, VRC requires user intervention to correct this error. This results in suboptimal combination without user supervision. The VBC method relies on compressing the data globally using a singular value decomposition (SVD) across the image. This can yield suboptimal combinations when completed at ultra-high fields [15, 16]. Thus, while these inline implementations are fast enough to be used for high resolution phase imaging, they tend to lack robustness and require user supervision [10, 17].

Post-acquisition combination methods require all the data to be collected before combination, making them difficult to apply to large datasets as they require the complex data from each coil to be exported, resulting in 32x to 64x larger amounts of data for typical studies performed with a head coil array. Common offline combinations include voxel-wise SVD [13], combining phase images from array coils using a short echo time reference scan (COMPOSER) [17], Block Coil Compression (BCC) [16], and the Adaptive Combine Phase Solution [18]. Voxel-wise SVD can be parallelized across voxels, but because all the processing must occur after acquisition is completed it would introduce significant processing delays if implemented inline for long time-series data such as fMRI. COMPOSER uses a specialized short echo reference prescan, and relies on scan-to-scan alignment which is completed using software such as FSL [19], which is not available on vendor-implemented reconstruction systems. Additionally, COMPOSER can result in edge effects such as Gibbs ringing when a low frequency prescan is used [20]. BCC uses a modification of the VBC method to initialize an ESPIRiT reconstruction [21] as ESPIRiT at ultra-high fields requires a locally varying phase estimate to capture the coil sensitivities. Unfortunately, BCC has high compute costs and would not be feasible on large datasets without refactoring. The adaptive combine phase solution [18] uses an SVD on a block of voxels to combine data with smooth image phase, but may not be optimal for ultra-high fields. These solutions all yield optimal or near optimal SNR combinations but are hard to implement for larger imaging datasets, such as time-series data.

One possible option to expand on existing offline coil combination methods is to use them on low resolution data to create a reference coil that can be applied to every imaging scan with minimal overhead. One potential method to generalize coil sensitivities from a low resolution prescan to higher resolution images is to fit them to a physically plausible basis. Previous work has shown that RF coil sensitivities are governed by the Helmholtz equations [22]. These equations rely on a wave number that is variable across the brain and can be difficult to estimate [23]. As an alternative, we suggest a relaxation of the Helmholtz equations whose solution is the solid harmonics. This basis is similar to the Helmholtz solution without the complexity of estimating a wavenumber. Fitting sensitivity profiles to the solid harmonics would allow them to be applied quickly to all images acquired during the imaging session.

**Fig 1:**
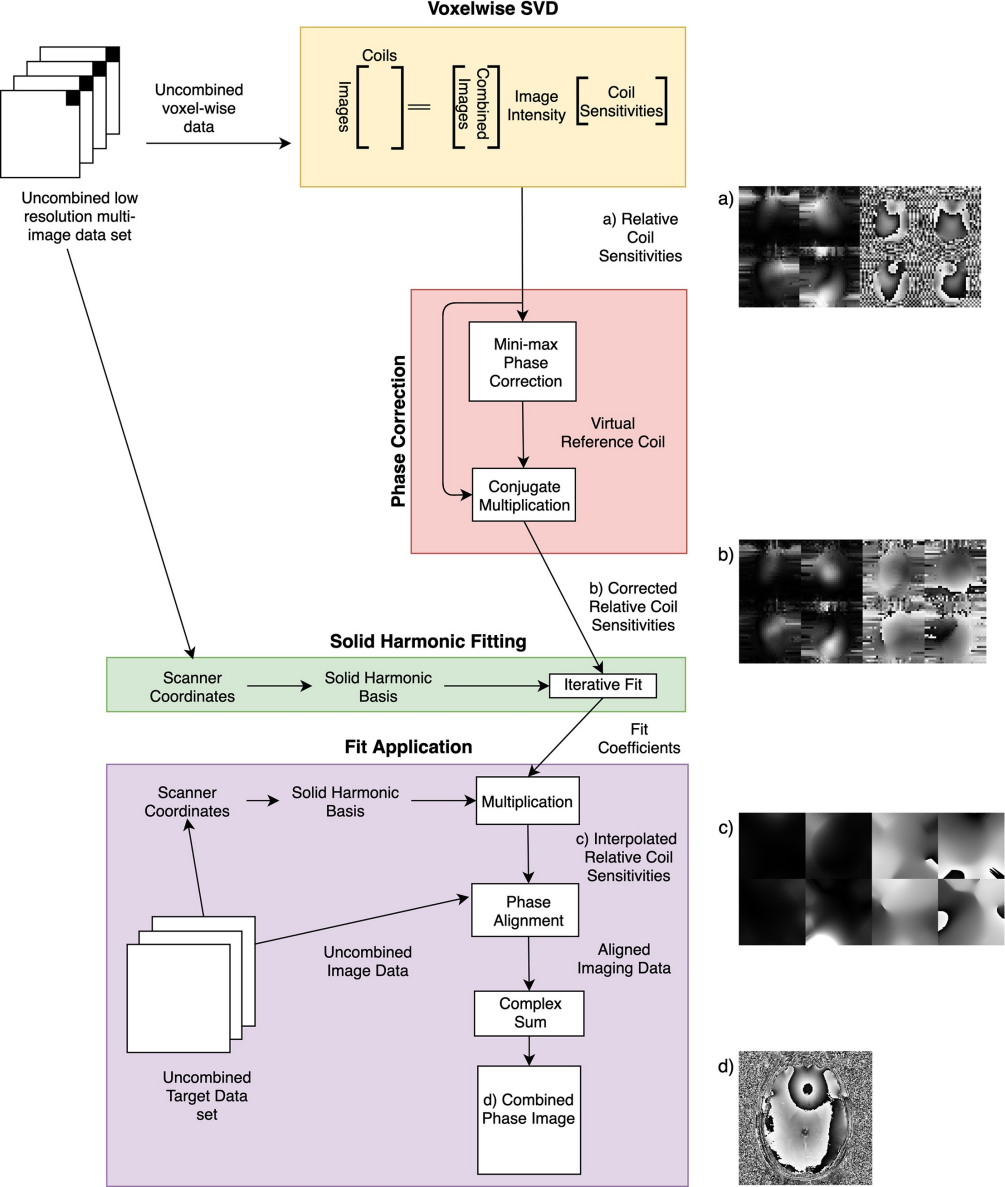
Flow chart of the fitted SVD method. Images represent example coil sensitivities across the same slice of the brain (four of 32 shown). The four left images are magnitudes of the coil sensitivities, and the four right images are phases of the coil sensitivities. a) Relative coil sensitivities calculated by voxel-wise SVD in prescan space, b) Coil sensitivities after alignment to a virtual reference coil created through minimax optimization across prescan space, c) Fitted coil sensitivities in target image space, d) Combined phase image after alignment with fitted coil sensitivities.

Coil combination of large imaging sets requires an inline method that is robust across the imaging session. Our proposed approach uses existing small, low resolution datasets to estimate coil sensitivities, in order to reduce the processing time requirements. These sensitivity estimates are then fit to a functional basis, allowing the estimates to be applied inline to any acquired geometry. Throughout the manuscript this method is referred to as the fitted SVD method and is outlined graphically in Fig 1. The fitted SVD method exploits the use of the routinely acquired $B_{1}^{+}$ shimming prescan on our parallel transmit (pTx) enabled 7T scanner in order to calculate relative receive coil sensitivities using a voxel-wise SVD. The use of SVD-derived sensitivities is similar to work done by previous groups that have used ESPIRiT [21], BCC [16] or the Adaptive Combine Phase Solution [18]. These relative receive coil sensitivities, as a consequence of the SVD algorithm, contain an arbitrary common phase which must be removed to allow accurate fitting. This common phase can be removed using a robust virtual reference coil [14] created through a minimax algorithm. The corrected relative coil sensitivities can then be iteratively fit to a physically plausible basis of solid harmonics to create a computationally efficient representation. The phase of these fitted coil sensitivities can be applied to align imaging data prior to complex sum combination to produce phase images. Hence, our proposed fitted SVD method is the amalgamation of ESPIRiT [21], voxel-wise SVD combination [13], VRC combination [14] and the Sbrizzi representation of sensitivities [22]. Combining these methods yields a technique tailored for robust acquisition of large multi-volume datasets for complex fMRI or fQSM, and additionally, may be applied to other acquisitions in the same session.

## Methods

### Mathematical methods

#### 1) Calculation of the relative receive coil sensitivities

The relative receive coil sensitivities can be calculated from a conventional voxel-wise SVD combination as follows. The measured complex-valued signal *s* from a voxel is given by the integral of the product of the receive coil sensitivity *c* and the magnetization *m* over the sensitivity volume of the voxel.


s=∫c(r)m(r)dV (1)

If either the coil sensitivities or the magnetization are assumed to be uniform over the integrated region, then the integral becomes separable and measured signal is given by the product of the average sensitivity $\bar{c}$ and the average magnetization $\bar{m}$.


$$s=\bar{c}\bar{m}$$ (2)

Assuming the coil sensitivity is constant in time, i.e., over multiple images, then a voxel’s measured signal for the *i*th coil and the *j*th image is given by

$$s_{i,j}={\bar{c}}_{i}{\bar{m}}_{j}$$ (3)

This can be represented as a rank one matrix **S**, where **c** is the vector of coil sensitivities and **m** is the vector of magnetizations across images, and *T* is the transpose operator.


$$S=cm^{T}$$ (4)

Assuming the noise in the measurements is uniform and normally distributed, the optimum least squares low rank approximation of **S** is given by the SVD [24], where the first left and right singular vectors give the best estimate of **c** and **m**, respectively. As singular vectors are defined to have unit norm, the magnitudes of **c** and **m** are contained in the first singular value, *λ*_1_:

$$\lambda_{1}={\left(c^{H}cm^{H}m\right)}^{1/2}$$ (5)

where *H* is the Hermitian conjugate. This deconstruction works with any number of coils and images greater than zero and is equivalent to the traditional sum of squares combination when only one image is used. However, the accuracy of **c** and **m** estimates is improved with increasing numbers of images. Estimation of **c** and **m** also improves with large variation in contrast in the images, such as in $B_{1}^{+}$ mapping.

#### 2) Phase correction of the relative receive coil sensitivities

The SVD of a complex matrix is only unique up to an arbitrary phase. Typically, the phase of the first element of the left or right singular vector is assigned to zero to impose a unique solution. If the phase of the right singular vector, i.e., **m**, is set to zero, this forces the phase of the magnetization for the first image, *φ*_**m**_, to be assigned to **c**. The estimated complex-valued coil sensitivity, **c**′, is then defined as:

$$c^{\prime }=c\frac{m_{1}^{H}}{{\left(m_{1}^{H}m_{1}\right)}^{1/2}}=\frac{c}{\left|c\right|}e^{{-i\varphi }_{m}}$$ (6)

*φ*_**m**_ comes from numerous sources, including the $B_{1}^{+}$ phase, off-resonance phase accrued from *B*_0_ inhomogeneities, and acquisition timing. It is preferable to set the phase of an image to zero because it is likely to be well defined over the entire imaging region, whereas the sensitivity of the first coil will often have areas where its magnitude approaches zero and the phase is therefore ill defined. Because *φ*_**m**_ contains $B_{1}^{+}$ contributions, it may contain phase singularities related to destructive interference during excitation. This is particularly an issue with parallel excitation schemes or ultra-high magnetic fields. These phase singularities introduced by *φ*_**m**_ do not correspond to magnitude nulls in the coil sensitivities and make solid harmonic fitting difficult. Therefore, it is necessary to remove *φ*_**m**_ from the coil sensitivity estimates. For a single voxel, any linear combination of **c**′ with weights, **w,** will also contain *φ*_**m**_ and can be applied to **c**′ to remove *φ*_**m**_ as follows:

$$\tilde{c}={\left(w^{H}c^{\prime }\right)}^{H}c^{\prime }={\left(w^{H}\frac{c}{\left|c\right|}e^{{-i\varphi }_{m}}\right)}^{H}\frac{c}{\left|c\right|}e^{{-i\varphi }_{m}}={\left(w^{H}\frac{c}{{\left|c\right|}^{2}}\right)}^{H}c$$ (7)

where $\tilde{c}$ represents the coil sensitivities with *φ*_**m**_ removed. Since **c** is desired, the optimum **w** would result in $\frac{w^{H}c}{{\left|c\right|}^{2}}$ being one. To maintain spatial phase coherence, the same **w** must be used for all voxels. Therefore, we want **w** that provides a spatially uniform virtual reference coil. We extend the voxel-wise case across the image by defining **C**′ as the matrix of all the relative sensitivities across all *k* voxels in the image as shown.


$$C^{\prime }=[{c^{\prime }}_{k}]$$ (8)

Finding **w** which makes $\frac{w^{H}c}{{\left|c\right|}^{2}}$ spatially uniform over all voxels is difficult and simply minimizing least-square deviation can lead to solutions with signal nulls which may remain in the final combination. These signal nulls are problematic because they introduce phase singularities common to all coils prior to fitting. This could result in phase singularities in the final image which will interfere with downstream processing. Alternatively, a robust elimination of signal nulls can be obtained via the use of a minimax algorithm which maximizes the minimum value of the combination across the image.


$$\underset{w}{\max}\;\underset{k}{\min}\left|w^{H}C^{\prime }\right|$$ (9)

This minimax estimation is restricted to the imaging volume by defining a SNR-based mask created via SNR threshold as discussed below in “Masking Considerations”. Using this method for finding **w** provides a non-uniform but signal null free **w**^*H*^**C**′ for removing *φ*_**m**_. The corrected relative coil sensitivities for all *k* voxels are

$$\tilde{C}=[{\tilde{c}}_{k}]$$ (10)

and is weighted by this minimax generated virtual reference coil.

#### 3) Fitting the relative receive coil sensitivities to a solid harmonic basis

In order to apply the relative coil sensitivities, it is necessary to represent them using a form that can be generalized to different orientations and resolutions. This can be done by fitting $\tilde{C}$ to a physically plausible basis in order to interpolate the estimated sensitivities. Such a basis set are the solid harmonics, which are composed of polynomial functions. If the coil sensitivities can be modeled as solid harmonics, $\tilde{C}$ can also be modelled using solid harmonics of higher order because it is the product of two coil sensitivities. A solid harmonic fitting basis is chosen for two reasons. First, solid harmonics are an efficient basis for spheroid shapes, such as human and animal heads. Secondly, the $B_{1}^{-}$ field, which governs the coil sensitivities, in a homogeneous medium follows the Helmholtz equation. The general solution of the Helmholtz equation is similar to the solid harmonics and therefore the solid harmonics are a physically plausible basis set that can be used to approximate the true $B_{1}^{-}$ field behavior.

Fitting to the solid harmonics is completed using variable exchange. After removal of *φ*_**m**_, $\tilde{C}$ will contain a virtual reference coil sensitivity component. This means that $\tilde{C}$ has an unknown common voxel-wise complex spatial scaling across coils, **d**. This common voxel-wise scaling originates from the minimax virtual reference coil and does not affect the relative phase of the individual relative coil sensitivities and its removal will only serve to improve interpolation quality. However, as **d** is only estimated, there could remain an incomplete removal of the virtual reference coil or physical common phase (such as $B_{1}^{+}$ phase) which may result in a low frequency spatial phase offset in phase images which will require background removal. As a result, this combination is best used for phase images for which further analysis uses phase differences [6, 9] or will employ postprocessing methods to remove low frequency background patterns [25]. Post processing to remove low frequency patterns would aid in correcting any asymmetry introduced into the image by incomplete removal of **d**.

The solid harmonic basis *A* is defined below where *r*,*θ*,*φ* represent the spherical coordinates, *N* is the maximal fit order, and $Y_{m}^{l}$ is a spherical harmonic

$$A(r,\theta ,\varphi )=\sum_{l=0}^{N}\sum_{m=-l}^{l}\sqrt{\frac{4\pi }{2l+1}}r^{l}Y_{m}^{l}(\theta ,\varphi )$$ (11)

The exchange is set up in two steps as follows:

$$min\sum_{i}{\left|AX-diag(d)\tilde{C}\right|}^{2}\;s.t.d^{H}d=k$$ (12)

Where ***X*** is the fit coefficients, $diag(d)=\left[\begin{array}{ccc} d_{1} & \cdots & 0\\ \vdots & \ddots & \vdots \\ 0 & \cdots & d_{k} \end{array}\right]$ and *k* is the number of voxels in the fit. The iteration begins with the calculation of **X** through least squares fitting. **d** is then calculated from $AX=diag(d)\tilde{C}$ and applied to the next iteration. The least squares fit was weighted by the square-root of the first singular value ($\sqrt{\lambda_{1}}$) in order to reduce the effects of noise in areas of low signal. The scaling of **d** is required to avoid the trivial **X** = 0, **d** = 0 solution. Fitting is completed over an SNR-based mask and is continued until the residuals of the least-squares fit change by less than 0.01%. The fit coefficients can then be used to estimate phase of the relative coil sensitivities and align receivers prior to combination.

#### 4) Image combination

The application of the relative sensitivity estimates can be done inline as each image is reconstructed. The complex signals are multiplied by the normalized conjugate of the relative sensitivity estimates and combined via a complex sum to create a complex image. This operation is applied voxel-wise as shown:

$$v=\frac{{\tilde{c}}^{H}}{\left|\tilde{c}\right|}s$$ (13)

The resulting phase of this image should be free of singularities and have high SNR. This could be further improved by inclusion of the noise covariance matrix if desired [12].

### Masking considerations

The fitted SVD method is reliant on masking out the regions without sensitivity information. To accomplish this, an SNR estimate was created using the ratio of the first and second singular values. This ratio is then thresholded by a hyperparameter in order to determine which voxels in the imaging volume should be included in either the minimax algorithm or the fitting. The effect of the threshold on the minimax algorithm was examined over nine values from 5 to 45. A second SNR threshold was applied during least squares fitting and was also tested over values from 5 to 45.

In order to apply the relative sensitivity estimates to images with differing geometries from the prescan it is necessary to constrain the fitted sensitivities to only parts of the image where the prescan data was able to estimate the sensitivities. Due to the known poor extrapolation performance of polynomial fits, a convex hull around all voxels used in the least squares fitting is computed. When applying the fit for phase alignment, voxels within the convex hull are aligned based on the fit and exterior points are aligned based on the fit at the closest point on the convex hull. This allows the method to be applied to differing fields of view and ensures only reliable coil sensitivity estimates are used.

### Imaging

All imaging was completed on the 68 cm bore 7T Siemens Magnetom Step 2.3 System equipped with an AC-84 Mark II head gradient coil located at the Centre for Functional and Metabolic Mapping at the University of Western Ontario. Imaging of three healthy volunteers (ages 23–27) was performed with written informed consent and approved by the Human Subjects Research Ethics Board at the University of Western Ontario. To investigate the fitted SVD method three datasets were acquired with one subject each: one dataset to compare the fitted SVD method to existing combinations, one dataset with an asymmetrical coil, and one dataset with subject motion.

#### Dataset 1: Comparative combination

This experiment used a whole head coil with a conformal 32 channel receive array and an eight channel transmit array operated in parallel transmit mode [26]. Three sets of images were acquired. First, prescan data was acquired for $B_{1}^{+}$ shimming which was then used as the low-resolution input for the fitted SVD method. This data consisted of one actual flip-angle imaging map [27] (TE/TR = 2.75/20 ms, FA = 70^o^) and 8 fourier encoded $B_{1}^{+}$ images [28] (TE/TR = 2.75/6 ms, FA = 5^o^) with resolution of a 8 mm isotropic, matrix size of 32x32x32, and BW = 1000 Hz/pixel. Next, an ultrashort echo time prescan was acquired to allow for comparison to COMPOSER [17], this data was a gradient recalled echo (GRE) with a resolution of 2x2x4 mm, matrix size 128x122x52, TE/TR = 0.8/5ms, FA = 10^o^, BW = 810 Hz/pixel and no acceleration. Finally, an acceleration free GRE sequence was collected 10 times. Five GRE images were used to generate high resolution coil sensitivities for testing the fitted SVD method on parameter matched data and five were used to calculate the voxel-wise SVD solution for the quality ratio calculation as well as serve as the target volume to combine when different combinations were compared. This target GRE sequence had a 1 mm isotropic resolution, matrix size 210x210x60, TE/TR = 7.7/15 ms, FA = 15^o^, BW = 140 Hz/pixel.

#### Dataset 2: Asymmetrical coil

This experiment used a highly asymmetric head coil with a conformal 32 channel receive array and eight channel transmit array also operated in parallel transmit mode, with both transmit and receive coils covering only the occipital-parietal regions [29]. This dataset consisted of two image sets, a $B_{1}^{+}$ prescan as described above and a gradient echo echo planar image set (GE-EPI) collected as the target image set to combine. The GE-EPI had a 2 mm isotropic resolution, matrix size 104x104x54, TE/TR = 20/1250 ms, FA = 45 ^o^, BW = 1457 Hz/pixel and GRAPPA factor 3 with 36 reference lines [11].

#### Dataset 3: Subject motion

This dataset was collected with a third coil that is the next generation whole head coil from the coil used for Dataset 1. It was a 32 channel receive array and eight channel transmit array with dipoles (rather than loops) as transmit elements and loops as receive elements [30]. As the dataset was investigating motion it was acquired in two parts. Part one consisted of a $B_{1}^{+}$ prescan and 5 GRE images without motion. Part two then instructed the subject to move in the coil before an additional GRE and another $B_{1}^{+}$ prescan were collected. This allows assessment of the fitted SVD method in the case of subject motion. Due to an intervening MRI system upgrade the prescan parameters are slightly different than the other two sets. The prescan data still consisted of one actual flip-angle imaging map [27] (TE/TR = 2.84/20 ms, FA = 50^o^) and 8 fourier encoded $B_{1}^{+}$ images [28] (TE/TR = 1.75/3.8 ms, FA = 3^o^) with a resolution of 8 mm isotropic, matrix size of 32x32x32, and BW = 1000 Hz/pixel. The target GRE sequence was collected identically to the target GREs in Dataset 1.

### Comparison metrics

To compare different combination techniques, three methods were employed. First, the output phase was unwrapped [31] and examined for singularities inside the volume of interest. Second, to quantify the performance of the fitted SVD method relative to other combinations, the quality ratio was measured across the target dataset for each combination method. The quality ratio is a measure of magnitude signal loss and therefore will be proportional to the phase SNR [32]. The quality ratio is defined as:

$$Q=\frac{\left|S_{method}\right|}{\left|S_{VSVD}\right|}$$ (14)

where *S*_*method*_ is the complex signal resulting from the combination method of interest and *S*_*VSVD*_ is the complex signal resulting from a voxel-wise SVD combination. This is a modification of the quality factor which uses the sum of the magnitudes in the denominator [10, 17]. The magnitude sum has a noise bias that is not present in the voxel-wise SVD combination. All average quality ratios are calculated across a brain mask excluding voxels less than 3% of the median value to reduce outliers such as large veins where signal is naturally too low to compare combination techniques [17]. Brain masks were generated based off sum-of-squares combined magnitude images using FSL’s Brain Extraction Tool (5.10.0) [19] and then eroded once using fslmaths. Finally, to compare the relative runtime of the different methods, all combinations were run single-threaded on a Centos 6.0 system with 256 GB of memory and Intel Xenon E5-2760 CPU and the reported runtime is the average clock time in seconds that the operation took to complete over five runs. This was performed single threaded as not all comparative combinations were available in a multi-threaded implementation.

### Fitted SVD parameter selection

Three input hyperparameters are required to use the fitted SVD method: the SNR-based mask thresholds for the minimax and least squares fitting steps as well as the fit order. In order to determine the optimal hyperparameter set in the case were the $B_{1}^{+}$ prescan is used to create sensitivities the fit was run from solid harmonic orders one to ten as well as nine equally spaced masking thresholds between 5 and 45 for both the minimax correction and solid harmonic fitting. The mean quality ratio and the coefficient of variation of the quality ratio were examined across the brain mask to determine the optimal hyperparameter set. The coefficient of variation is defined as:

$$CV=\frac{\sigma }{\mu }*100$$ (15)

Where *σ* is the standard deviation over the brain mask and *μ* is the mean quality ratio. The mean quality ratio determines what degree of signal loss that a parameter set incurs but the coefficient of variation ensures that the spread in quality ratios is consistent across the brain.

### Comparative combinations

Complex sum, voxel-wise SVD [13], VRC [14], COMPOSER [17], and the fitted SVD method were all implemented using in-house MATLAB code (R2018a) that is available at: https://gitlab.com/ostanley1/phasecombofunctions-matlab. These are also briefly described below. The BCC method [16] was implemented using the toolbox provided by the authors.

At the time of development, complex sum was the default on the MRI system for functional phase data (CMRR-MB on the Siemens scanner [11] prior to R16 (2017)). The CMRR-MB versions prior to 2017, when this approach was first developed, used a simple sum of all the coil data followed by a calculation of the phase. The CMRR Multiband EPI sequence on Siemens systems after 2017 is not known to have any of the issues considered in this paper. We were particularly interested in this sequence for studying the phase effects of large vessels in fMRI, but the approach we describe is applicable to all types of images, where appropriate phase combinations may still not be available. Voxel-wise SVD [13] is completed by calculating the SVD of a matrix formed by volumes and coils. To prevent singularities resulting from the arbitrary phase of the SVD, the phase of the first volume is set to zero making this method a measure of relative phase as opposed to absolute phase.

The VRC method [14] uses a voxel as a reference to align the coil images and create a reference coil. The reference voxel is chosen as the voxel with the largest minimum magnitude across all coils [14]. This voxel’s phases are then subtracted from each coil profile before summation to create a virtual reference coil. This virtual reference coil is then subtracted from each coil profile to create phase offsets which are smoothed with a three dimensional 10mm gaussian blur and used to align the data prior to combination.

COMPOSER [17] was implemented using the FSL registration tool FLIRT (5.10.0) on the magnitude images to determine the transformation between the short echo time reference image and the target data. Uncombined coil data was then saved to real and imaginary NIFTIs and this transformation was applied to both the real and imaginary components separately [19]. These transformed reference images were used to remove shared coil signal prior to image combination using complex sum.

The BCC method [16] uses a regional SVD to create a common reference coil block by block, followed by aligning adjacent blocks to ensure phase smoothness. Once this reference data is created the data undergoes an ESPIRiT combination [21] using the newly created virtual coil as a reference channel to ensure successful phase combination.

The fitted SVD method was completed on the $B_{1}^{+}$ shimming dataset and a set of five matched scans identical to the target image set. This was done to examine the effects of using a prescan for fitting and to compare against a reference approach using identical parameters to the target image set. The method was developed to use a multi-image prescan such as the $B_{1}^{+}$ shimming datasets because they are routinely collected on pTx systems and can be used with no additional imaging time requirements. On non-pTx systems other multi-image sets could be used such as those collected for B_0_ shimming to obtain the same time benefits.

### Temporal noise

To compare the noise across time the EPI data from Dataset 2 was used. The fitted SVD method was used to calculate sensitivities from the $B_{1}^{+}$ prescan and was applied to each volume in the EPI series. As comparators, the VRC and BCC sensitivities were calculated from the first volume and applied to every volume in the series and voxel-wise SVD was performed across all volumes. Finally, the fitted SVD method was performed using the sensitivities from the voxel-wise SVD as input, a case equivalent to performing the fitted SVD method on matched image data. Once these time series were created the phase of the first volume was removed and the images were unwrapped through time to remove any jumps of 2π. In order to remove system drift, the time series were linearly detrended voxel-wise prior to calculating the phase noise. The temporal standard deviation was then calculated to create phase noise images. The phase noise ratio between each combination and the voxel-wise SVD was used to investigate differences in phase noise levels between combinations. It is defined as:

$$Phase\;Noise\;Ratio=\frac{\sigma_{method}}{\sigma_{VSVD}}$$ (16)

where *σ*_*method*_ is the temporal standard deviation of the phase time course for the combination method of interest and *σ*_*VSVD*_ is the temporal standard deviation of the phase time course for the voxel-wise SVD. Comparing noise relative to a reference method removes sources of variance shared across combination methods such as an increase in noise in lower SNR areas of the asymmetric coil. This was done across a brain mask with voxels less than 3% of the median removed as in the quality ratio comparison.

## Results

### Fit order and masking threshold selection

**Fig 2:**
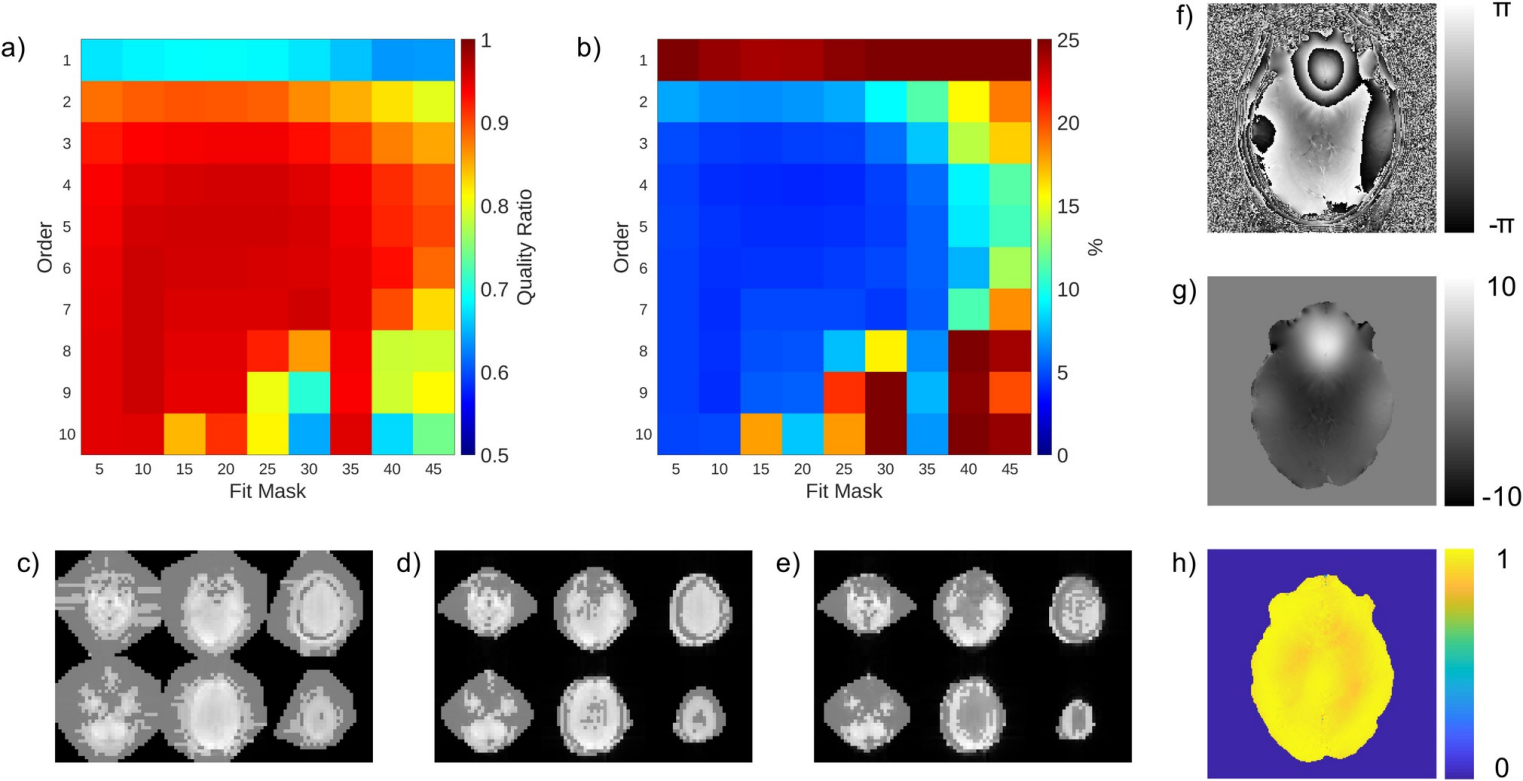
Fitted SVD method in a human using $B_{1}^{+}$ prescan data from a single subject. a) Average quality ratio and b) coefficient of variation of quality ratio as a function of fit order and fit mask size. Example convex hull (grey) and voxels included in fit (white) for various mask thresholds c) 10, d) 20, e) 30. f) Example phase image, g) unwrapped phase image, and h) quality ratio map at the selected parameters (order 6, fit mask of 20, minimax mask of 20).

The fitted SVD method relies on three hyperparameters: the thresholds for the SNR-based masks during minimax phase correction and solid harmonic fitting as well as the order of the solid harmonic basis. The effect of solid harmonic order and SNR-based masking during the fit are shown in Fig 2 for a single subject. To assess performance the quality ratio was averaged over an eroded brain mask generated using FSL’s BET tool on all sixty slices (S1 Fig). These results show there is a large parameter space which allows for high quality combinations. For this paper the chosen parameters were SNR-based mask thresholds of 20 for the minimax algorithm and 20 for the least squares fitting and a basis of solid harmonic order 6 which yields a high mean quality ratio of 0.96±0.04 (µ±σ) and a low coefficient of variation of 4.4%. This shows that using a low resolution prescan slightly reduces phase SNR (4% reduction), but still effectively combines the data.

### Fitted SVD and comparative methods

**Fig 3:**
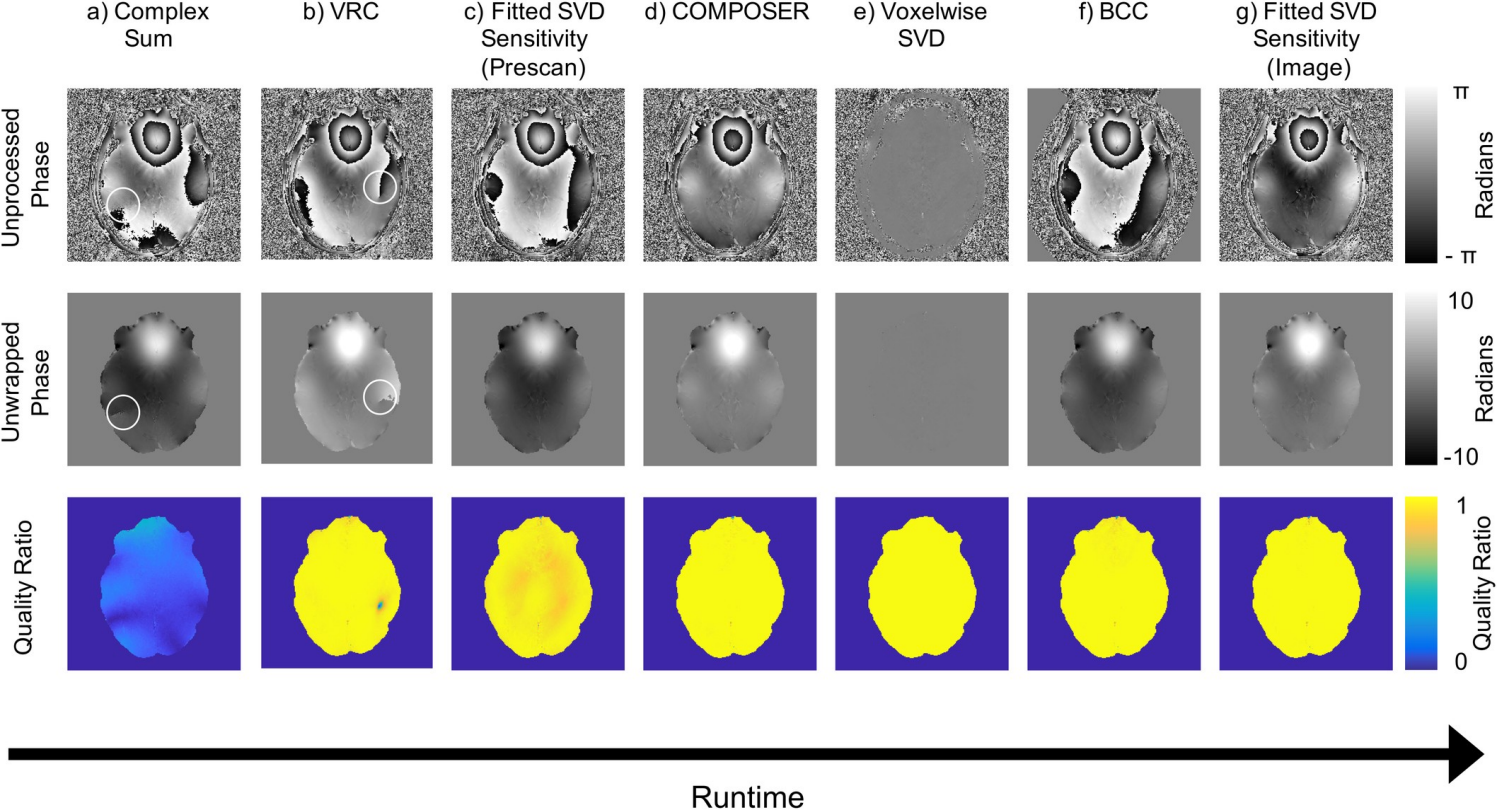
Comparison of phase combination methods. One example slice is shown for each method. Top row: raw phase image, Middle row: unwrapped phase image for easier visualization (singularities circled in white), Bottom row: quality ratio across a representative slice. a) Complex sum combination, b) VRC c) Fitted SVD method using a low resolution $B_{1}^{+}$ prescan, d) COMPOSER, e) Voxel-wise SVD combination, f) BCC, g) Fitted SVD method using parameter matched dataset. Single threaded runtime of each method increases left to right and can be found in Table 1. Note: the BCC method applies a rough mask to the region-of-interest during combination and this causes zeros in the exterior of the raw phase image.

To investigate the quality of the fitted SVD method against other benchmarks, three criteria were used: singularities, quality ratio, and runtime. Fig 3 shows a qualitative comparison between complex sum, VRC, the fitted SVD method using prescan data, COMPOSER, BCC, voxel-wise SVD combination, and the fitted SVD method using a parameter matched image set. Receiver-based phase singularities can indicate destructive interference, the worst case of coil combination, and any phase combination method should not produce these artifacts. Singularities present in the complex sum are corrected in all the combination methods except the VRC method. Singularities can also be present due to global phase shared across coils and in this case still present post processing difficulties that need to be corrected. This was the case in the VRC combination where it was not possible to obtain an acceptable virtual coil for the VRC method using the maximum shared signal method for voxel selection [14]. The voxel selected was outside the brain in our target data and produced a reference with signal nulls and phase singularities (S2 Fig). Unfortunately, this is not a robust option for phase combination as the singularity introduced by the reference coil will cause downstream processing issues when the data is further analyzed. For the fitted SVD method, the minimax algorithm was used to overcome this inherent VRC limitation. One additional observation is that most methods do result in a left-right asymmetry that can be seen in the wrapped and unwrapped phase images. The two exceptions appear to be BCC and COMPOSER. The images in Fig 3 are sorted by relative runtime. One consideration when comparing runtimes is that fitted SVD method runtimes include both relative receive sensitivity estimation and fitting as well as applying the fit to the target dataset. The estimation of the fitted sensitivities needs to only be done once per session and then can be applied to the remaining images in the session. This application of the fitted sensitivities takes 18 seconds on the target data when the prescan was used for fitting.

Phase singularities represent complete signal loss at their location in the image however there can be subtler SNR decreases throughout the brain. To identify phase SNR decreases it is necessary to compare quality ratio between combination methods (Fig 3 and Table 1). The fitted SVD method can combine the target image with no loss of phase SNR when matched resolution images are used. In contrast, there was a slight quality degradation (4%) when the lower resolution $B_{1}^{+}$ prescan data was used. This degradation was small compared to the complex sum combination. Although methods such as COMPOSER and BCC show fractionally higher quality ratios, this is offset by substantially larger computational expense which makes using them for large phase datasets impractical.

**Table 1:** Summary of coil combination methods quality and single threaded runtime when implemented in Matlab R2018a.

Combination Method	Singularities Present	Quality ratio (mean±std)	Runtime in Matlab (seconds)
Complex Sum	Yes	0.17±0.08	0.09
Virtual Receive Coil	Yes	0.98±0.05	1.2
Fitted SVD Method (Prescan data)	No	0.96±0.04	36
COMPOSER	No	1.00±0.03	137
Voxel-wise SVD	No	1.00±0.00	400
Block coil combination	No	1.00±0.04	2700
Fitted SVD Method (Image data)	No	1.00±0.03	4900

All quality ratio values are calculated over the entire brain mask.

### Fitted SVD and the occipital-parietal coil

**Fig 4:**
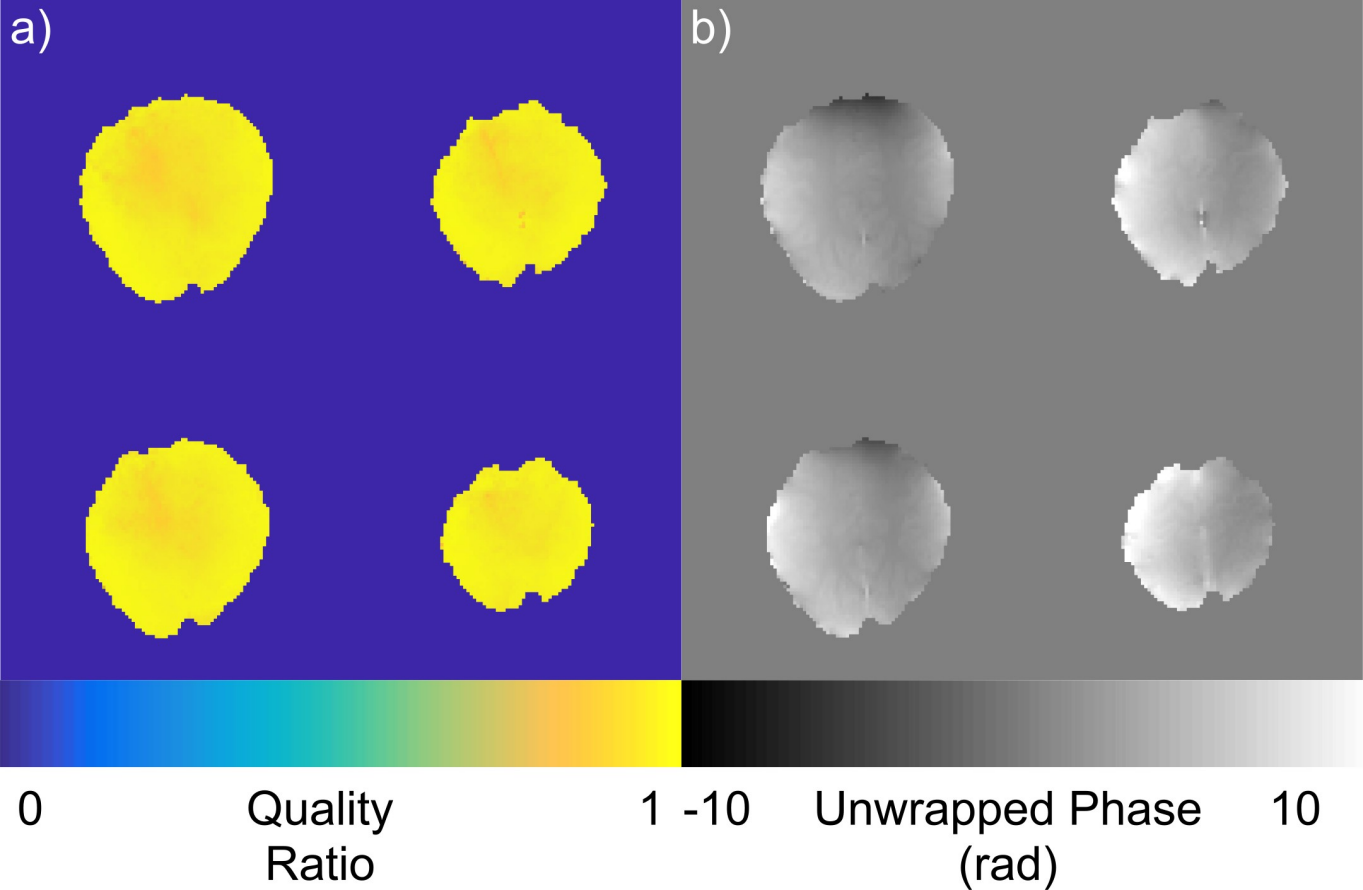
Combination quality of an asymmetrical coil. a) Quality ratio of data collected in an occipital parietal coil when combined with the fitted SVD method, b) Spatially unwrapped phase data after fitted SVD combination.

To investigate potential coil geometry dependency of the fitted SVD method, it was used with an occipital-parietal coil designed for high-resolution imaging of the visual system [29]. The same fit parameters were used from the whole head coil. The combination shows no degradation in signal in the areas targeted by the coil (Fig 4). The quality ratio across the area of interest was 0.95±0.04 when the $B_{1}^{+}$ prescan was used to determine coil sensitivities. As this combination was done without new parameter selection for the occipital-parietal coil, this demonstrates that the solid harmonic fitting is not dominated by RF receiver design and the fitted SVD method can operate even when imaging with an asymmetrical coil.

**Fig 5:**
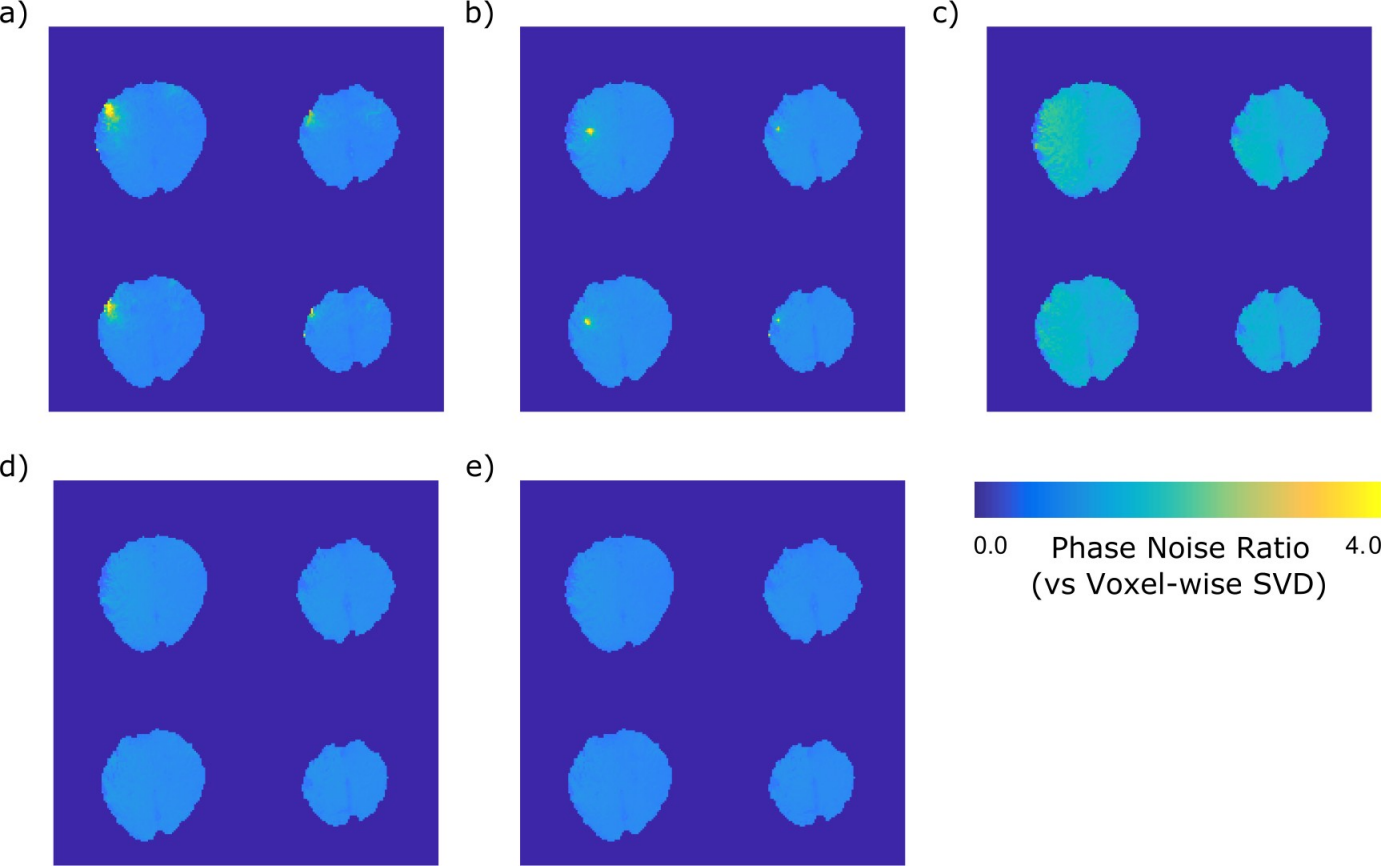
Phase noise ratios in an asymmetrical coil. Voxel-wise SVD was used as the reference method. Phase noise ratio combined using a) complex sum, b) VRC, c) BCC, d) the fitted SVD method using the B1+ prescan, and e) the fitted SVD method using the EPI timeseries as input. Hyperintensities correspond with phase singularities in a and b.

The functional data acquired using the occipital-parietal coil also allowed for investigation of the phase noise over time. This was investigated by calculating the temporal standard deviation of the unwrapped and linearly detrended phase time courses to create phase noise images. The ratios of these phase noise images were then calculated between each combination and, our reference method, the voxel-wise SVD (Fig 5). Two combination methods lead to singularities in the combined images when the EPI data was used (Fig 5A and 5B) and these can be seen in the noise images as hyperintensities. BCC shows elevated phase noise throughout the image (Fig 5C). The phase noise ratio images show that there are no large increases in noise between voxel-wise SVD and the fitted SVD method using a prescan (Fig 5D) or a matched image set (Fig 5E), demonstrating that for applications such as complex fMRI using the fitted SVD method will not lead to significant additional noise. This is advantageous because using voxel-wise SVD can become expensive when operating on long timeseries or large image sets.

### Fitted SVD and subject motion

**Fig 6:**
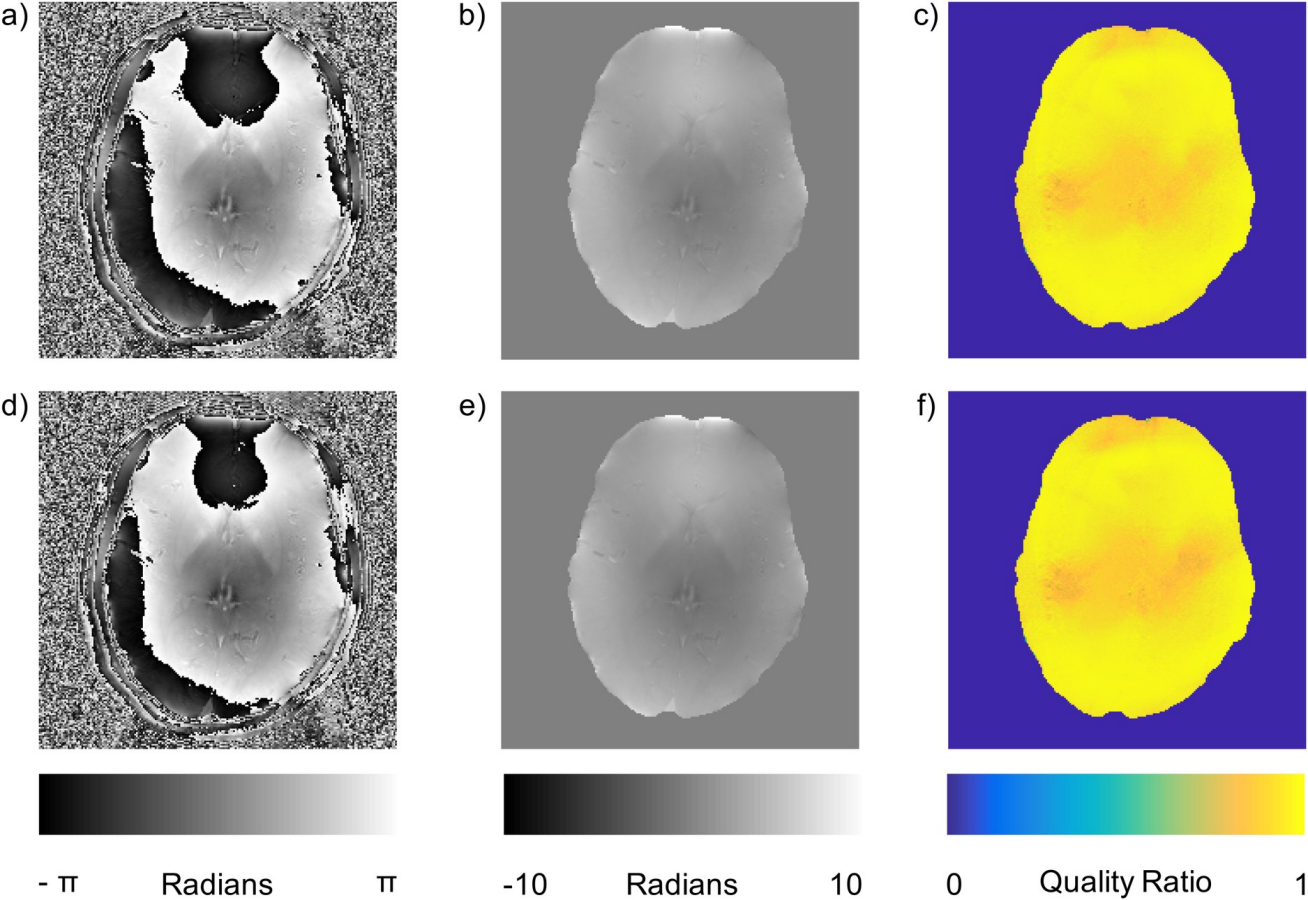
Effects of motion on the fitted SVD method. a) Raw phase image, b) unwrapped phase image, c) quality ratio map created with no motion between the $B_{1}^{+}$ prescan and the imaging. d) Raw phase image, e) unwrapped phase image, f) quality ratio map created with 3.5 mm motion between the $B_{1}^{+}$ prescan and the imaging. No singularities were observed.

Finally, it is necessary to investigate the fitted SVD method in the case of subject head motion. Subject head motion could slightly change the coil loading and as a result could degrade the quality of the phase combination as the sensitivities change. This limitation is always a concern when using any prescan based approaches, including reference lines for accelerated acquisitions. A $B_{1}^{+}$ prescan and five target GREs were collected after which the subject was asked to move in the coil and a single GRE and the $B_{1}^{+}$ prescan were collected again. Registration between the first GRE and the reference collected after the subject moved show the subject had a root-mean-squared motion of 3.5 mm, far beyond the tolerance of any functional study and representing a true worst case scenario with respect to subject motion [33]. The premotion $B_{1}^{+}$ prescan resulted in a quality ratio across the brain of 0.95±0.05. When the prescan collected after large head motion was used the quality ratio remained the same (0.95±0.05). This demonstrates that combination quality is tolerant of significant head motion (Fig 6). This is likely due to the smooth spatial frequency characteristics of the solid harmonic fitting and the low resolution prescan.

## Discussion

Phase imaging requires robust coil combination to be useful. In large multi-volume imaging datasets, such as those acquired for fMRI or fQSM, inline combination becomes vital as the computational load for exporting uncombined data can be prohibitive (hours for a typical fMRI timeseries). The fitted SVD method was created to combine these large imaging sets, though it is equally applicable for all MRI applications. Its implementation will allow for combination of phase data inline, expanding the utility of phase based image processing such as fQSM [9] or phase regression at high resolution [8]. This method is needed as these applications are growing fastest at high field strengths where the combination issues are most pronounced. The idea of creating a phase combination method tailored to a specific application has already been established in the literature. Several methods have already been established for multi-echo data for QSM such as phase difference methods [34], ASPIRE [35] and voxel-wise SVD combination (Eq 1, [13]). In addition, work has been undertaken to complete a reference free coil combination of water fat imaging [36] and provide a bias free combination for QSM [20]. The proposed fitted SVD method is another such approach to optimizing phase sensitive combination to a specific application, in this case large functional imaging datasets. This method is uniquely suited to processing large datasets in two ways: (1) by creating a combination that could be applied to the data during inline reconstruction and (2) by ensuring the method is robust across coil configurations and motion.

### The fitted SVD method will require no export of data off system

In order to avoid export of large uncombined datasets off the MRI system, the coil sensitivities could be quickly estimated using prescans and then applied to a scanning session during inline reconstruction. This is future work. However, in MATLAB testing, the use of a B_1_^+^ prescan reduced computational runtime by two orders of magnitude compared to using data with parameters matched to the target imaging set. When compared to other combination methods, the fitted SVD method performed faster than BCC or COMPOSER [17] and had no singularities, like VRC [14] or complex sum (Table 1). These results establish that this method is a suitable trade-off between quality and functionality.

### The fitted SVD method is robust across coil configurations and motion

Several features of the fitted SVD method were designed to increase its robustness for routine use. First, the use of a voxel-wise SVD to derive receive coil sensitivities makes the method extensible to any multi-image prescan, including several of the conventionally used shimming prescans. As a result, this method easily fits into existing protocols and produces images with a quality ratio of 0.96±0.04 (mean±std) when using these prescans, as opposed to 1.00±0.03 when using a matched image set. The image created by the default combination on our MRI system results in a quality ratio of 0.17±0.07 across the brain and contains phase singularities (Fig 3). Second, use of the minimax algorithm to create a virtual receive coil increases robustness above maximum shared signal selection [14], making this method more robust than VRC. Finally, through fitting the coil sensitivities to a basis, we can extend their utility to scans of various geometries with minimal SNR penalties (Figs 2 and 4). This fitted SVD method produces a stable combination across time (Fig 5) as well as maintains high SNR results in the case of extreme subject motion (Fig 6). The solid harmonics can model sensitivities from a coil with symmetrical or asymmetrical geometry to produce high quality ratio images (Fig 4). As the solid harmonic solution is a relaxation of the Helmholtz equations, this method should also be able to model coil sensitivities far from the head, where the shapes are non-spheroid [22], although more investigation is required. These factors demonstrate that the fitted SVD method is a robust phase sensitive combination.

### Applications of the fitted SVD method for phase combination

This fitted SVD method can be used for any type of imaging and is ideally positioned to combine large multi-volume datasets such as those used in complex valued fMRI and fQSM. Although the fitted SVD method results in a left right asymmetry this is not due to a reduced quality ratio and can either be corrected using relative phase across time [6] or high pass filtering as is common in QSM [20]. Several other factors make it attractive for phase combination in other applications, including that this method can be applied in absence of a body coil, making it a strong choice in a research environment that uses high *B*_0_ fields where body coils cannot be used to estimate coil sensitivities. Additionally, implementation of the method requires no extra acquisitions in a conventional pTx scanning protocol, due to the use of an existing prescan to derive the receive coil sensitivity estimates. This combination method can be used for all applications free of supervision, as the three parameters governing its operation can be set to be optimal for the specific system (and potentially coil) as needed. In this study, once these parameters were selected, there was no case collected in the three datasets in which the fitted SVD method produced singularities. These factors make the fitted SVD method useful for any high field system in need of a push button solution, particularly those applications that acquire multivolume phase data.

### Study limitations

This preliminary study of the fitted SVD method used datasets targeted at the goal application for analysis. These experiments included our proposed target application, functional phase imaging, and provided estimates of quality as well as temporal noise. Future work could further investigate the efficacy of this technique across a larger subject group to ensure quality in other applications.

The quality of the fitted SVD method does depend on the resolution of the data used to derive coil sensitivities, which becomes a trade-off between quality versus time because the low-resolution nature of the prescan reduces the time required to fit it to the solid harmonics. The fitting will take more time if it is applied to higher resolution data, but this trade-off does not result in a large phase SNR decrease (4%, Fig 2). This method is also limited by any motion between the prescan and the imaging session, however the worst case analysis in our head coil shows that this effect is minimal (Fig 6). In fact, the resistance of this method to motion makes it an excellent candidate for functional imaging. While it may not be suited to every application of phase imaging, it is an excellent option for those that would otherwise be limited in compute resources.

## Conclusions

In conclusion, the fitted SVD method proposed in this pilot study potentially allows for robust phase coherent combination inline and with minimal phase SNR loss. This method is an extension of the existing ESPIRiT, voxel-wise SVD, and VRC combination methods. Using voxel-wise SVD allows us to compute coil sensitivity estimates from routinely acquired prescans without relying on a physical reference coil. Using a minimax optimization to determine our virtual reference coil has removed shared singularities from our sensitivities and ensures a good fit across the region of interest. The solid harmonic fitting allows us to use the power of the voxel-wise SVD combination on a small, acquired dataset and apply that solution to align and combine the entire session for better phase imaging that takes full advantage of conventionally acquired protocols. These different steps allow for stable phase imaging on high throughput systems such as ultra-high field research systems, allowing for phase contrast images to be added without additional scan or compute time.

## Supporting information

S1 Fig All slices of Dataset 1 for inspection for artifacts. a) phase image, b) unwrapped phase image, and c) quality ratio map at the selected parameters (order 6, fit mask of 20, minimax mask of 20).
(TIF)

S2 Fig VRC combination. a) Image of the largest minimum magnitude across all coils for VRC reference voxel selection. Voxel is in red and is indicated by a red arrow. b) Virtual reference coil created when using the selected voxel. A singularity is circled in red. This singularity is also present in the combined images and using VRC in this case results in an image with a phase singularity which affects downstream processing.
(TIF)
