# Supplementary figures and images for: Receiver phase alignment using fitted SVD derived sensitivities from routine prescans
Source: PLoS One. 2021 Aug 30;16(8):e0256700. doi: 10.1371/journal.pone.0256700 (PMC8404984; doi:10.1371/journal.pone.0256700)

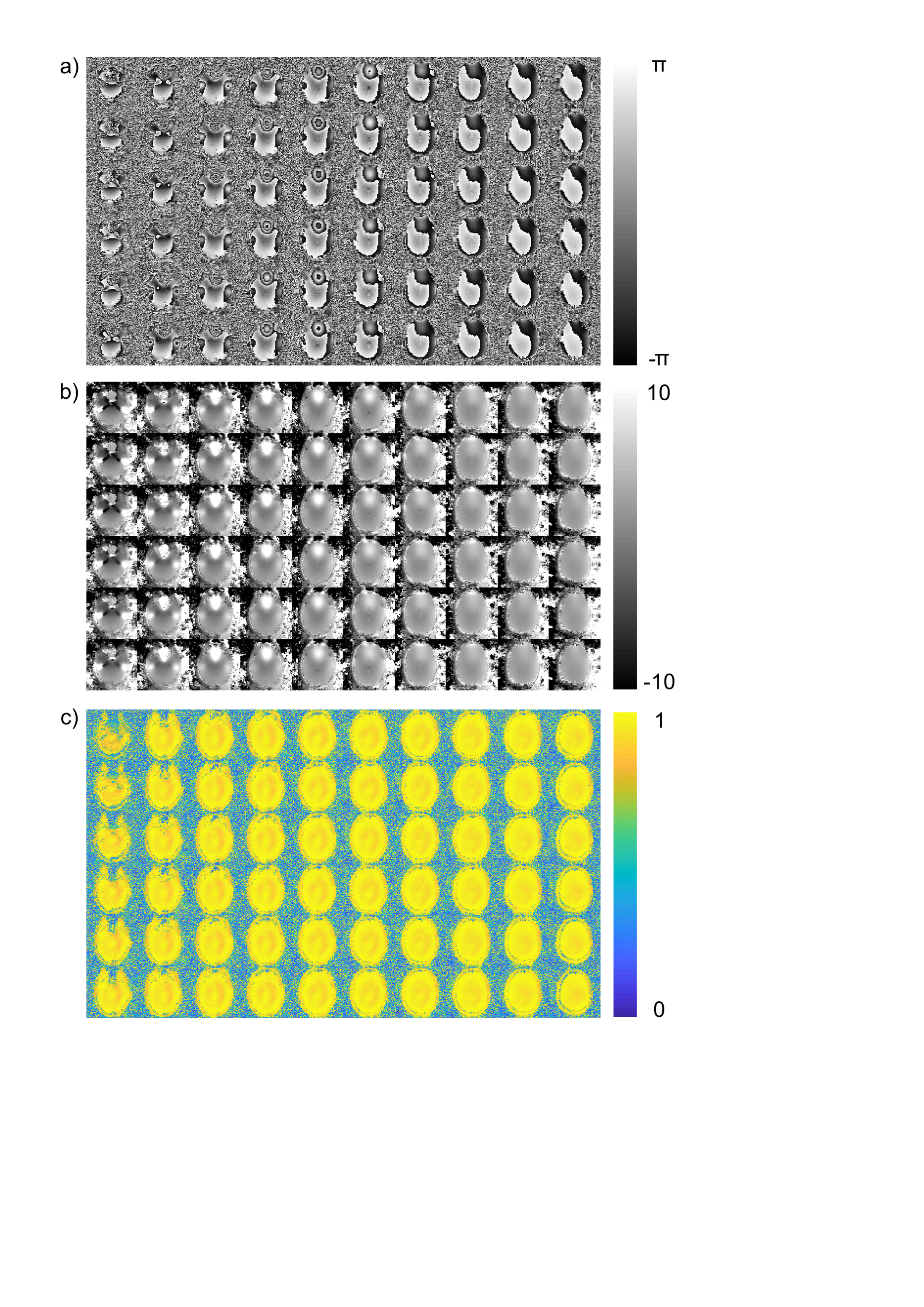

Supplement: S1 Fig — a) phase image, b) unwrapped phase image, and c) quality ratio map at the selected parameters (order 6, fit mask of 20, minimax mask of 20). (TIF) [file pone.0256700.s001.tif]

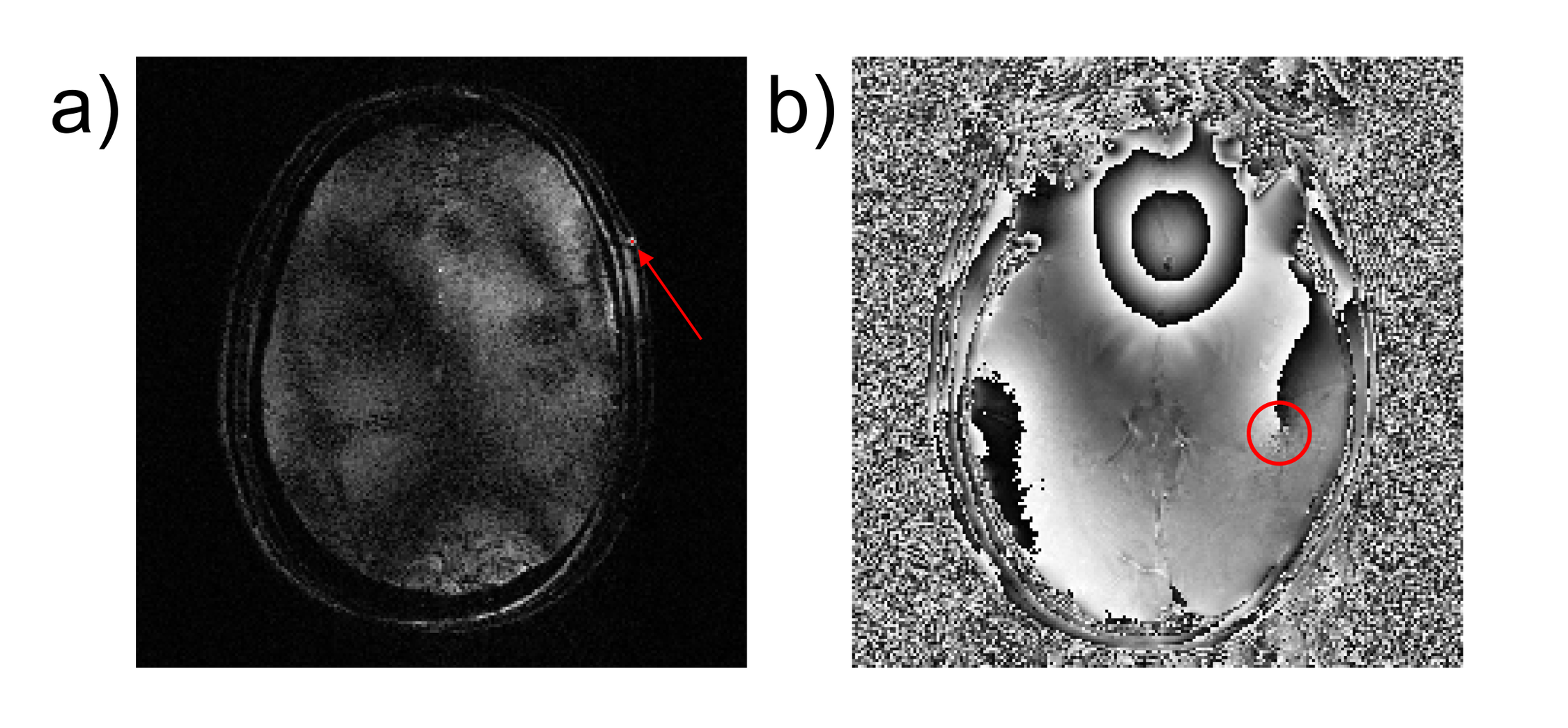

Supplement: S2 Fig — a) Image of the largest minimum magnitude across all coils for VRC reference voxel selection. Voxel is in red and is indicated by a red arrow. b) Virtual reference coil created when using the selected voxel. A singularity is circled in red. This singularity is also present in the combined images and using VRC in this case results in an image with a phase singularity which affects downstream processing. (TIF) [file pone.0256700.s002.tif]
